# Chlorin Endogenous to the North Pacific Brittle Star *Ophiura sarsii* for Photodynamic Therapy Applications in Breast Cancer and Glioblastoma Models

**DOI:** 10.3390/biomedicines10010134

**Published:** 2022-01-08

**Authors:** Antonina Klimenko, Elvira E. Rodina, Denis Silachev, Maria Begun, Valentina A. Babenko, Anton S. Benditkis, Anton S. Kozlov, Alexander A. Krasnovsky, Yuri S. Khotimchenko, Vladimir L. Katanaev

**Affiliations:** 1 Institute of Life Sciences and Biomedicine, Far Eastern Federal University, 690922 Vladivostok, Russia; klimenko.am@dvfu.ru (A.K.); rodina.ee@dvfu.ru (E.E.R.); begun.ma@dvfu.ru (M.B.); khotimchenko.ys@dvfu.ru (Y.S.K.); 2 A.N. Belozersky Research Institute of Physico-Chemical Biology, Moscow State University, 119899 Moscow, Russia; proteins@mail.ru (D.S.); babenkova@belozersky.msu.ru (V.A.B.); 3 Federal Research Center of Biotechnology of the Russian Academy of Sciences, 119071 Moscow, Russia; anton93benditkis@yandex.ru (A.S.B.); anton4ikk_06@mail.ru (A.S.K.); phoal@mail.ru (A.A.K.); 4 Translational Research Center in Oncohaematology, Department of Cell Physiology and Metabolism, Faculty of Medicine, University of Geneva, 1211 Geneva, Switzerland

**Keywords:** porphyrin, chlorin, singlet oxygen, photodynamic therapy, ophiura, cancer, breast cancer, glioblastoma, mouse models

## Abstract

Photodynamic therapy (PDT) represents a powerful avenue for anticancer treatment. PDT relies on the use of photosensitizers—compounds accumulating in the tumor and converted from benign to cytotoxic upon targeted photoactivation. We here describe (3*S*,4*S*)-14-Ethyl-9-(hydroxymethyl)-4,8,13,18-tetramethyl-20-oxo-3-phorbinepropanoic acid (ETPA) as a major metabolite of the North Pacific brittle stars *Ophiura sarsii*. As a chlorin, ETPA efficiently produces singlet oxygen upon red-light photoactivation and exerts powerful sub-micromolar phototoxicity against a panel of cancer cell lines in vitro. In a mouse model of glioblastoma, intravenous ETPA injection combined with targeted red laser irradiation induced strong necrotic ablation of the brain tumor. Along with the straightforward ETPA purification protocol and abundance of *O. sarsii*, these studies pave the way for the development of ETPA as a novel natural product-based photodynamic therapeutic.

## 1. Introduction

Photodynamic therapy (PDT) relies on the use of photosensitizers—compounds that are relatively benign until excited by light of a particular wavelength that converts them into an activated state resulting in the generation of reactive oxygen species [1,2,3]. PDT finds multiple applications in medicine, particularly in anticancer therapy, where it has been approved to treat cancers in the skin (basal cell carcinoma), lungs, or esophagus [2,3]. Other forms of cancer, such as in the breast or brain (e.g., glioblastoma) have so far evaded approved PDT applications [2,3].

A number of photosensitizers have been marketed for PDT in different countries, and the search for novel compounds never ceases [3,4]. Photosensitizers activatable in the red part of the spectrum are particularly sought, as the red light can penetrate deeper in biological tissues [1,3]. Derived from chemical synthesis, these complex compounds weigh upon the costs of PDT; stability in body fluids is another issue with synthetic photosensitizers [3,4].

Natural products have always been one of the major sources of new drugs, including oncology therapeutics [5,6,7]. Porphyrin-type compounds such as chlorins can act as efficient photosensitizers and have been found in diverse living groups [4,8]. In a search for novel anticancer compounds from ophiuras [9], we have recently discovered the chlorin (3*S*,4*S*)-14-Ethyl-9-(hydroxymethyl)-4,8,13,18-tetramethyl-20-oxo-3-phorbinepropanoic acid (ETPA) from a North Pacific brittle star *Ophiura sarsii*—the first-ever porphyrin identified in Ophiuroidea (phylum Echinodermata) [10]. This discovery was unexpected, as Ophiuroidea were believed to lack porphyrin/chlorin synthesis [11] and raised the hypothesis that the *Ophiura sarsii* chlorin was the result of dietary (and perhaps seasonal) consumption by these marine invertebrates [10].

In the current work, we provide evidence that ETPA production is endogenous to *Ophiura sarsii* and is independent of their food consumption. As a major metabolite in this abundant brittle star species and amenable to simple purification, the 663 nm light-absorbing chlorin shows sub-micromolar phototoxicity against a panel of cancer cells in vitro and serves as an efficient PDT against glioblastoma in a mouse model. Our findings pave the way for the development of ETPA as a natural photosensitizer in a broad spectrum of PDT applications.

## 2. Materials and Methods

### 2.1. Species Collection and Food Deprivation

Brittle stars *Ophiura sarsii* were collected at the depths of 15–18 m near the Vyatlin Cape (Russky Island) in the Peter the Great Gulf, Sea of Japan, in December 2020. Sample collection was performed by the standards approved by the Ministry of Science and Higher Education (Russia); all efforts were made to minimize animal suffering. Brittle stars (wet weight—25 g) were separated into two groups. The first group, after thorough 2× running water rinsing, was frozen and stored at −80 °C. The second was placed into a clean spacious aquarium regularly refilled with fresh sterilized and filtered seawater without any source of food. Gradual emptying of the animals’ digestive tracks could be visually seen in the first days of such fasting. After 12 days, the food-deprived animals were rinsed and stored as the first group.

### 2.2. Homogenization and Extraction

*O. sarsii* were mechanically homogenized and then sequentially placed in solvents with increasing polarity: hexane, chloroform, ethanol, and water (125 mL each) at room temperature for 8–12 h, with constant stirring on a PSU-10i shaker (Biosan, Riga, Latvia). The ratio of solvents to the homogenized mass was 5:1 (volume). The resulting extracts were cleared through paper filters and concentrated on a Hei-VAP Value Rotary Evaporator (Heidolph, Schwabach, Germany) at 37–40 °C under vacuum (chemical vacuum station PC 3002 VARIO (Vacuubrand, Wertheim, Germany)). The chlorin (3*S*,4*S*)-14-Ethyl-9-(hydroxymethyl)-4,8,13,18-tetramethyl-20-oxo-3-phorbinepropanoic acid (ETPA) localized to the ethanol fraction [10] that contained 721.5 mg (control brittle stars) and 579 mg (fasted brittle stars) dry weight. To remove salts from the ethanol extract, liquid-liquid extraction was performed. The ethanol extract was dissolved in 30 mL n-butanol and 50 mL water and transferred to a separatory funnel. The organic (butanol) phase was extracted 3× with 50 mL water. Then, n-butanol was removed from the fraction using the rotary evaporator, followed by dissolution of the precipitate in methanol for subsequent chromatography.

### 2.3. Analytical and Semi-Preparative HPLC 

Analytical and Semi-Preparative HPLC was performed on a Shimadzu system (Shimadzu, Kyoto, Japan) equipped with LC-20AP modular pumps, an SPD-20A spectrophotometric detector, and an FRC-10A fraction collector. Analytical separation was performed on a Shim-pack SHIMADZU GIST C18 column (250 mm × 4.6 mm, particle size 5 μm). MeOH was used as the mobile phase solution A, and water acidified with 0.1% formic acid—as solution B. Chromatography was performed at a rate of 0.6 mL/min at 40 °C, with a maximum pressure of 10 MPa. Elution was achieved with a gradient: from 50 to 95% MeOH solution in 55 min, then 95% solution for 10 min, then returned to 50% MeOH in 5 min. Reanalysis was performed after complete conditioning of the column. Detection was carried out on a spectrophotometric detector at wavelengths of 210 nm and 366 nm.

Separation of extracts was carried out on a Shim-pack SHIMADZU GIST C18 column (250 mm × 10.00 mm, particle size 5 μm) with a mobile phase of methanol (solution A) and water acidified with 0.1% formic acid (solution B). Chromatography was performed at a rate of 2.6 mL/min at 23 °C. The gradient was from 80 to 97% solution in 50 min, 97% solution A in 8 min, then return to 80% MeOH in 7 min with UV detection at 210 nm and 366 nm.

### 2.4. High-Resolution Spectrophotometry 

High-Resolution Spectrophotometry of the chromatographic fraction containing ETPA dissolved in methanol was performed by a UV-1800 spectrophotometer (Shimadzu, Kyoto, Japan) in the wavelength range from 200 to 800 nm.

### 2.5. Cells and Medium

Human breast cancer cells MCF-7, BT-20 and MDA-MB-231 (all from ATCC, atcc.org), rat C6 glioma cells and human embryonic kidney (HEK-293 cells, both from Collection of vertebrate cell cultures, Institute of Cytology, Russian Academy of Sciences (incras.ru/wp-content/uploads/2019/06/katalog_rccc_v_2018_rus.pdf, accessed on 21 December 2021) were cultured in DMEM + GlutaMAX medium (Gibco, Waltham, MA, USA) supplemented with 10% fetal bovine serum (Biosera, Nuaille, France) and 1% antibiotic–antimycotic (Gibco, USA). Cell cultures were grown in a CO_2_ incubator with a Galaxy 48R cell vitality monitoring and vitality system (Eppendorf, Hamburg, Germany) at 37 °C and 5% CO_2_.

### 2.6. Photoxicity Assays

ETPA was dissolved in DMSO. In 96-well microculture plates, human breast cancer and glioma cells were seeded at the density of 3000 cells/well and cultured overnight in a CO_2_ incubator at 37 °C. After medium removal, 50 μL DPBS with serial dilutions of ETPA was added for 2 h (the resultant concentration of DMSO in the wells was <0.2%). Cells in pure DPBS served as a positive control; wells with DPBS without cells served as a negative control. Next, the cells were irradiated with red light in the wavelength range from 580 to 780 nm using a 2000–4000 lux LED lamp for 30 min. Removal of ETPA immediately before light exposure did not reduce/influence the resulting phototoxicity. Photosynthetic photon flux density (PPFD) was measured in 12 randomly chosen wells using the LI-190R Quantum Sensor (LI-COR Biosciences, Lincoln, NE, USA) and found to vary from 217–580 μmol/m^2^/s. Fluence was measured (using a Newport Optical Power Meter 842-PE, MKS Instruments, Norwood, MA, USA) to be 16.04 J/cm^2^. Next, a 200 μL culture medium was added to each well for additional incubation for 72 h in a CO_2_ incubator at 37 °C.

To assess cell death, the culture medium was removed and 50 μL of MTT (triazolyl blue tetrazolium bromide) reagent (DIA-M, Moscow, Russia) dissolved in DPBS at 0.5 mg/mL was added for 3 h incubation at 37 °C. After aspiration of the liquid, 100 μL DMSO was added to each well for 5 min before measuring the optical density at wavelengths of 570 and 630 nm using a Cytation 5 multifunctional plate reader (BioTek, Winooski, VT, USA). MTT was separately performed for C6 and HEK-293 cells in the dark to obtain light-independent cytotoxicity. IC_50_ and standard error of the mean (SEM) were obtained by standard dose–response curve fitting using GraphPad Prism 8.

### 2.7. Determination of the Singlet Oxygen Quantum Yield of ETPA

Singlet oxygen was detected using two methods. One of them was based on the measurement of the rates of chemical trapping of singlet oxygen by 1,3-diphenylisobenzofuran (DPIBF) (Acros Organics, Geel, Belgium, >99%) as described [12,13]; see Supplementary Figure S1. Having a strong absorption maximum at 414 nm, DPIBF is efficiently oxidized by singlet oxygen forming colorless products having no absorption maxima in the visible spectral region. The rate of DPIBF bleaching is directly proportional to the rate of ^1^O_2_ production by irradiation of a photosensitizer. The absorption spectra of the trap solutions were recorded with the SF-56 spectrophotometer (LOMO Spektr, St. Petersburg, Russia). For irradiation, a xenon lamp and grating monochromator were employed.

Another method was based on detection of the infrared phosphorescence of singlet oxygen at 1270 nm, which arises due to the energy transfer from the triplet state of the photosensitizer molecules to oxygen, followed by the population of singlet oxygen (the reactive excited singlet (^1^Δ_g_) state of oxygen molecules); see Supplementary Figure S2. Measurements were carried out using a laser/LED spectrometer assembled at the Federal Research Center of Biotechnology of the Russian Academy of Sciences [14]. The spectrometer allowed phosphorescence detection upon excitation by pulses of LED with the emission maxima at 399 nm (Polironik, Moscow, Russia). Phosphorescence was recorded at a 90^o^ angle with respect to the excitation beam through the cut-off filter that transmitted IR light at λ > 1000 nm and one of three interchangeable interference filters with transmission maxima at 1230, 1270, and 1310 nm and half-width of 10 nm. The photodetector was an FEU-112 photomultiplier (Ekran Optical Systems, Novosibirsk, Russia) (PMT), with the S-1 spectral response cooled to −35 °C. PMT impulses were sent to a broadband (0–200 MHz) preamplifier and then to a USB computer board, which was launched by additional electric pulses synchronous with the pulses of the LED. The signal of the board was processed by a personal computer with the Parsec (Dubna, Russia) software. As a result, the time interval between pulses was divided into 256, 512, or 1024 channels, and the computer showed the number of PMT impulses accumulated in each channel during the irradiation time, thus forming the kinetic curves of singlet oxygen phosphorescence after LED pulses.

The phosphorescence method provides more information on singlet oxygen than the trapping method. However, the trapping method is much more sensitive. Acetone was employed as the solvent for the singlet oxygen measurements because ETPA is readily soluble in it. The absorption spectrum of ETPA in acetone is shown in Supplementary Figure S3.

### 2.8. Mouse Experimentation

Mouse Experimentation was conducted in accordance with the ethical standards and recommendations for accommodation and care of laboratory animals covered by the Council Directives of the European community 2010/63/EU on the use of animals for experimental studies. The animal protocols were approved by the institutional animal ethics committee of A.N. Belozersky Research Institute of Physico-Chemical Biology, Approval Code: Protocol 8/21, Approval Date: 7 September 2021.

### 2.9. Cell Culture and Intracranial Tumor Implantation

C6 glioma cells were harvested with trypsin/versene while in the logarithmic phase of growth before intracranial stereotaxic implantation as a single cell suspension (1 × 10^6^ cells/mL) into young C57BL/6 female mice (18 ± 3 g). Under isoflurane anesthesia (2–2.5% in air) by the SomnoSuite^®^ system (Kent Scientific Corporation, Torrington, CT, USA), the mouse was placed in a stereotactic frame, and the skull was exposed through a midline incision cleared of connective tissue and dried. Implantation was performed at the following coordinates: ML, −2.5; AP, −1.0; DV, −3.0, as previously described [15]. C6 glioma cells (5 × 10^5^ per mouse) were implanted with Robot Stereotaxic (Neurostar, Tubingen, Germany) using a Hamilton microsyringe at the speed of 3 μL/min in 10 µL PBS.

### 2.10. Glioma Photodynamic Therapy (PDT)

PDT was performed 7 days after intracranial tumor implantation. Anatomical positioning of the tumor was obtained by brain magnetic resonance imaging (MRI) visualization. Tumor-bearing mice were sensitized via intravenous injection into the jugular vein of ETPA at 40 mg/kg of body weight 6 h pre-PDT; the drug dose regime was chosen following the study by [16]. Application of ETPA and PDT were conducted under isoflurane anesthesia. The brain skull in the illumination area was thinned with a milling cutter and illuminated for 30 min with a red laser light source (L04-1H, 650 nm, output power 100 mW) producing a 1.5 mm diameter light beam, positioned on the region corresponding to the stereotaxic coordinates of the prior tumor injection. The surface of the skull was constantly cooled with saline to avoid thermal damage to the brain. Rectal temperature was kept constant at 37.0 ± 0.2 °C using a heating pad. After PDT, the animals were returned to cages, provided with water and food ad libitum, and continually monitored for any signs of neurological deficit. The tumor-bearing group included 5 mice.

### 2.11. Magnetic Resonance Imaging and Histological Studies of the Tumor Injury

PDT-induced tumor injury was identified by analyzing brain MRI scans obtained 5 days after PDT on a 7-T magnet (Bruker BioSpec 70/30 USR; Bruker BioSpin, Ettlingen, Germany) using an 86 mm volume resonator for radiofrequency transmission and a phased array mice head surface coil for the reception. Before scanning, the animals were anesthetized with isoflurane 2–2.5% in a mixture of oxygen and air. Mice were placed in a prone position on a water-heated bed. The heads of the mice were immobilized using a nose mask and masking tape. The imaging protocol included a T2-weighted image sequence (time to repetition = 4500 ms, time to echo = 12 ms, slice thickness = 0.5 mm). After MRI, the animals were sacrificed, and the brains were removed, fixed, sectioned, and stained by hematoxylin–eosin.

## 3. Results

### 3.1. Chlorin Is Endogenous to O. sarsii

**Figure 1:**
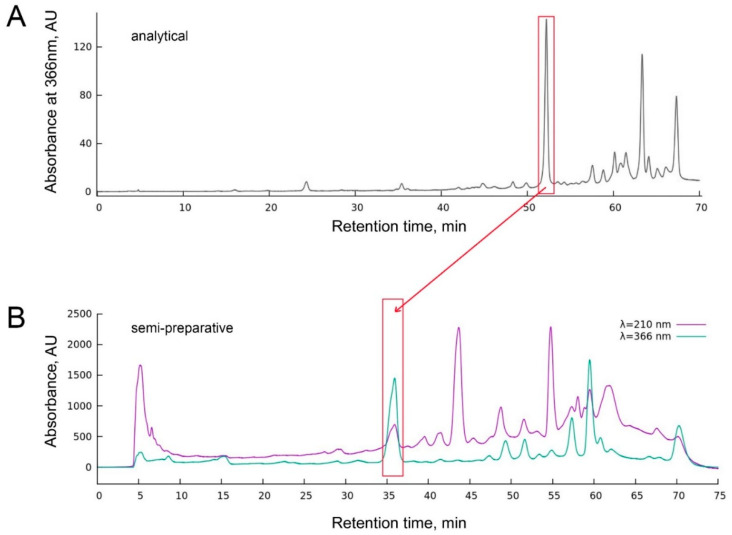
Analytical (**A**) and semi-preparative (**B**) chromatography of the butanol fraction of the EtOH extract of *O. sarsii*, absorption intensity at the wavelength of 366 nm (**A**) and 210 nm/366 nm (**B**). The major peak at the retention time of 52 min (**A**) and 35 min (**B**) corresponds to ETPA (chlorin) from our previous study [10]. Chromatograms from non-fasted ophiuras are shown in both panels; samples from the fasted ophiuras show identical chromatograms.

Since Ophiuroidea as the class of the Echinodermata phylum were considered porphyrin-free [11], we hypothesized that the chlorin (3*S*,4*S*)-14-Ethyl-9-(hydroxymethyl)-4,8,13,18-tetramethyl-20-oxo-3-phorbinepropanoic acid (ETPA) we previously discovered in the North Pacific *Ophiura sarsii* could result from the dietary consumption; the resulting seasonal variability in the ETPA content was also considered possible [10]. To address this issue, we performed a new *O. sarsii* collection in the same location but another season (December vs. May in [10]). Analytical HPLC (Figure 1A) of the butanol fraction of the ethanol extract of the freshly collected *O. sarsii* contained a major peak at a wavelength of 366 nm at the retention time of 52 min, which corresponds to ETPA as the major 366 nm absorbing compound from our previous study [10], arguing against a seasonal diversity in the new chlorin compound in the brittle stars.

In order to directly rule out that ETPA could be a dietary derivative of *O. sarsii*, we separated the fresh catch of the brittle stars into two portions, one subjected to direct freezing and processing and the other fasted for 12 days prior to processing for preparative isolation (see Methods). Briefly, 15 mg and 20 mg of the butanol fraction of the EtOH extracts from non-fasted and fasted brittle stars, respectively, were subjected to semi-preparative chromatography (Figure 1B). Identical chromatograms were obtained for fasted and non-fasted preparations, with the major 366 nm absorption peak (retention time 34–37 min), corresponding to ETPA from our prior work [10], collected for subsequent analyses.

**Figure 2:**
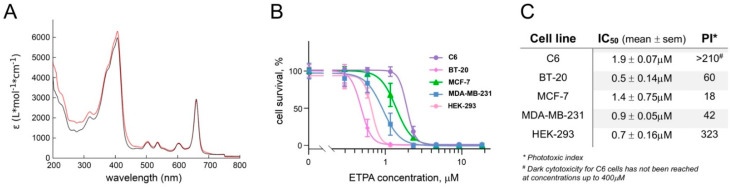
(**A**) Absorbance spectra of the chlorin compound isolated from the butanol fraction of the EtOH extract from control (red) and fasted (black) *O. sarsii* reveal absorbance peaks typical for chlorins, identical between the two preparations; (**B**) cell survival (MTT test) to evaluate the strong phototoxicity of ETPA after red-light irradiation. Data are given as mean ± SEM, n = 8 (3 for HEK-293 cells); (**C**) ETPA phototoxicity IC_50_ and the phototoxicity index (PI, calculated as IC_50_ in the dark/IC_50_ in the light [17]). Cell survival data in the dark are taken from [10] (breast cancer cell lines) or measured separately (C6 and HEK-293 cells, see Supplementary Figure S4).

To control the identity of the compound in the collected fractions, spectrophotometry was performed in the wavelength range of 200–800 nm. The resulting absorbance spectra were identical for the compound isolated from the fasted and non-fasted brittles stars and revealed the absorption typical for chlorins, with the absorbance peaks at 204, 293, 409, and 663 nm (Figure 2A), also fully coinciding with ETPA isolated by us from *O. sarsii* previously [10].

The final yield of the chlorin compound was 0.56 mg from the non-fasted and 0.6 mg from the fasted ophiuras, or 3.7% and 3%, respectively. With these essentially identical yields, and with the lack of seasonal variability in the chlorin content, we concluded that ETPA is not part of the dietary preferences of the brittle stars but is endogenously synthesized by them.

### 3.2. Phototoxicity of ETPA against a Panel of Cancer Lines

The chlorin (ETPA) isolated from *O. sarsii* has shown dark cytotoxicity against a panel of breast cancer cell lines, with IC_50_s in the range of 25–45 µM [10]. In order to assess whether the anticancer effect could be increased upon illumination of the compound to grant potential applicability for PDT, we next studied the phototoxic effect of ETPA.

Breast cancer cells lines BT-20, MCF-7, and MDA-MB-231, along with the glioma cell line C6 and non-cancerous HEK-293 cells, were preincubated for 2 h with increasing concentrations of ETPA before irradiation with a red-light LED lamp (580 to 780 nm) for 30 min (fluence = 16.04 J/cm^2^). Cell growth in the subsequent 72 h was assessed with the MTT assay (see Methods). Resulting data (Figure 2B,C) show striking phototoxicity of ETPA, with the sub-micromolar to low-micromolar IC_50_s and impressive phototoxic indices (PI, measured as IC_50_ in the dark/IC_50_ in the light [17], Figure 2C), arguing for the strong potential of this natural chlorin in PDT applications. Notably, dark phototoxicity for the glioma C6 cells was not achieved at the highest concentrations of ETPA tested (Figure 2C and Supplementary Figure S4).

### 3.3. Singlet Oxygen Production by ETPA

**Figure 3:**
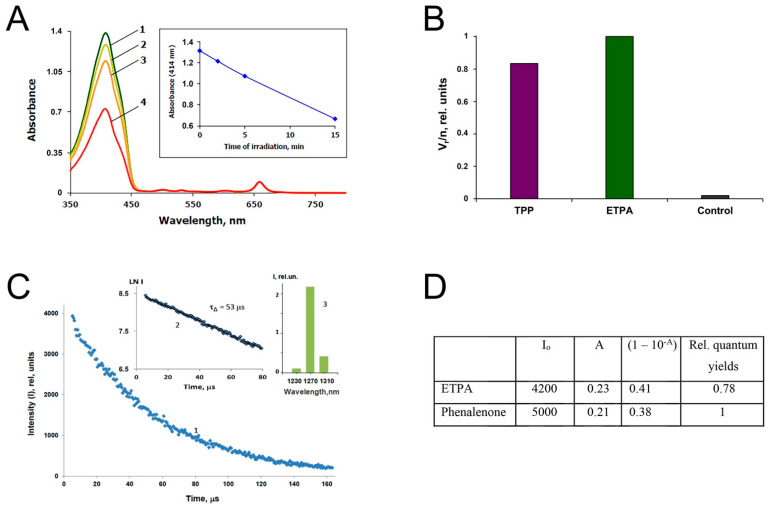
ETPA efficiently generates singlet oxygen: (**A**) changes in the absorption spectrum of DPIBF (curves 1–4) in the mixture of DPIBF with ETPA in acetone during irradiation by monochromatic red light (660 nm) absorbed by ETPA. Here, “2” corresponds to 2 min, “3” refers to 5 min, and “4” to 15 min irradiation. Power of exciting light was 83 μW. Inset shows the time course of 414 nm absorption fall of DPIBF. ETPA bleaching was not observed; (**B**) the relative quantum yields of DPIBF oxidation (V_r_/n) upon irradiation of TPP and ETPA. The rate of spontaneous DPIBF bleaching in the dark without sensitizer and irradiation is defined as “Control”. For TPP, irradiation time was 10 min, excitation wavelength was 512 nm, irradiation power was 105 μW, and absorbance of TPP at 512 nm was 0.024. For ETPA, irradiation time was 2 min, excitation wavelength was 660 nm, excitation power was 83 μW, and absorbance at 660 nm was 0.095; (**C**) kinetic trace of photosensitized phosphorescence of singlet oxygen upon excitation of ETPA by 5 μs pulses of violet LED (399 nm) in cartesian (1) and semilogarithmic (2) coordinates. Pulse repetition rate was 5 kHz, average LED power was 30 mW, and irradiation (averaging) time was 10 min. The PMT signal was accumulated using a time-resolved computer photon counting. The duration of one channel was 640 ns, and the number of channels was 256. Absorbance of the solution at 396 nm was 0.246 in a 1 cm quartz cell. Here, “3” indicates phosphorescence emission spectrum estimated using three interchangeable interference filters. I corresponds to the phosphorescence intensity just after the end (5 μs) of the LED pulse. The decay time (τ_Δ_) of the phosphorescence exactly coincided with the known value of singlet oxygen lifetime in acetone; (**D**) calculation of the quantum yield of singlet oxygen by ETPA in comparison with phenalenone.

Two methods to measure singlet oxygen production by ETPA upon illumination were employed (see Methods). The first was based on the chemical trapping of singlet oxygen by 1,3-diphenylisobenzofuran (DPIBF) and bleaching of DPIBF at 414 nm upon ETPA irradiation. The ETPA excitation was produced by the monochromatic 660 nm red light corresponding to the ETPA absorption maximum, which is not absorbed by DPIBF (Figure 2A and Supplementary Figure S3). Figure 3A shows the time-dependent decay in DPIBF 414 nm absorbance upon red-light illumination in the presence of ETPA. When comparing the efficiency of ETPA with that of meso-tetraphenylporphyrin (TPP, Figure 3B) known to produce singlet oxygen with the quantum yield of 0.7 [12,13], the absolute quantum yield of singlet oxygen generation by ETPA was determined as 0.83. Taking into consideration the relative error for such measurements at ±10% of the average value, the singlet oxygen yield for ETPA can be estimated as 0.8 ± 0.1 from the trapping experiment.

The second method was based on detection of the infrared phosphorescence of singlet oxygen (see Methods), comparing the phosphorescence intensities (Figure 3C) in solutions of ETPA and phenalenone—one of the most efficient photosensitizers of singlet oxygen generation, with the quantum yield of this process close to one [14]. For calculations, so-called zero-time intensities (I_o_) of phosphorescence were used, which were obtained by extrapolation of the semilogarithmic kinetic plots to the zero time. The obtained I_o_ values were then normalized to the absorption coefficients (1–10^−A^) of the pigments at the wavelength of excitation. The resulting data are summarized in Figure 3D. 

Thus, both methods indicate that the quantum yield of singlet oxygen production by ETPA is close to 0.8, identifying ETPA as very a strong photosensitizer of singlet oxygen generation with promising for biomedical applications.

### 3.4. Photodynamic Therapy with ETPA in a Mouse Model of Brain Tumor

**Figure 4:**
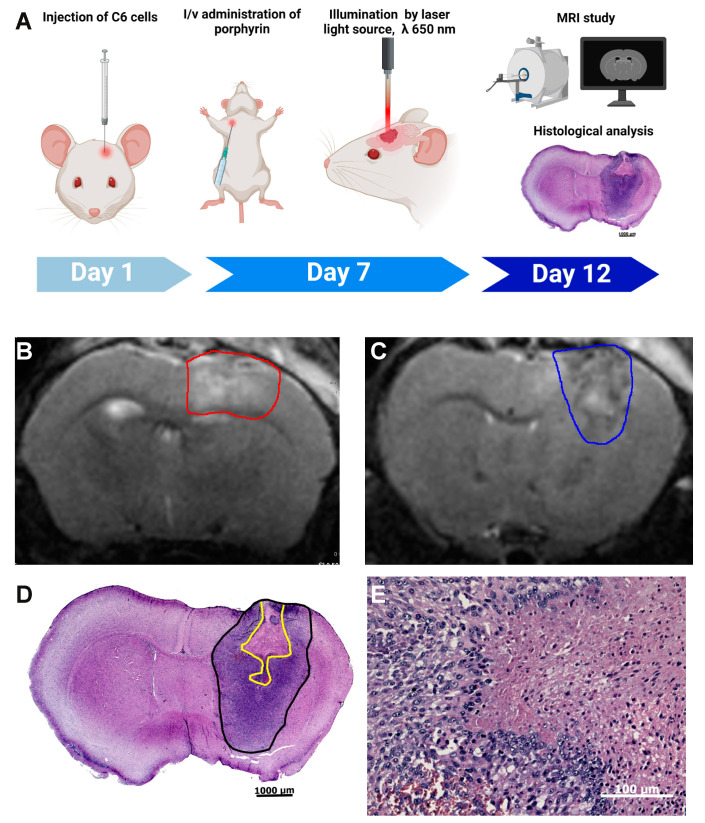
PDT using ETPA in a mouse model of glioblastoma: (**A**) scheme of the experiment; (**B**,**C**) representative T2-weighted MR images from coronal brain sections (0.5 mm thick) obtained 5 days after PDT. The area outlined with a red line refers to hyperintensities regions (edema, **B**). The area outlined with a blue line refers to PDT-induced glioma necrosis (**C**); (**D**) a representative histological section, stained with eosin–hematoxylin, demonstrates the tumor boundaries (outlined in black) and the presence of necrotic loci in the area of laser illumination (outlined in yellow); (**E**) enlarged area showing the boundaries of necrotic and intact tumor tissues. Images shown are representative of 3 animals.

Inspired by the strong phototoxicity of ETPA against a panel of cancer cell lines in vitro, and by the especially high phototoxic index of our compound against glioma cells (Figure 2C), we next aimed at performing in vivo experiments of anticancer PDT with the brittle star-derived chlorin compound. Glioblastoma is the most common form of brain tumor, characterized by low responsiveness to treatment and poor prognosis (median survival < 2 years) [18]. Rat glioma C6 cells simulate human glioblastoma when injected into rats [19], and mouse brains [20,21] and have been a popular model in glioblastoma research [22]. We implanted C6 glioma cells in mouse brains (see Methods). After 7 days, IV administration of ETPA was performed, 6 h prior to brain MRI-guided targeted tumor illumination with a red laser (650 nm, see Methods and Figure 4A).

Five days post PDT (Figure 4A), brain anatomical and histological analyses were performed. Through brain MRI, analysis of T2W-images revealed a strong signal change in the area exposed to laser irradiation. Figure 4B illustrates that in the tumor area exposed to laser irradiation, a hyperintense signal prevails indicating accumulation of water molecules in this area, i.e., tissue edema as the result of the photodynamic tissue damage. In the central part of the tumor (Figure 4C), the signal is mixed with zones of the hyperintense signal. Further histological analysis confirmed that this area corresponds to the area of necrosis (Figure 4D). Most of one hemisphere is occupied by a tumor consisting of poorly differentiated fusiform cells with large nuclei and narrow cytoplasm; many mitotic cells can also be seen. The stroma is poorly expressed. In the center of the tumor, there is an area of necrosis (a hypereosinophilic region devoid of nuclei), in the form of a pyramid with a broken apex. The area of necrosis occupies approximately 15% of the area of the tumor tissue in the section. At the border between dead and living tissue (Figure 4E), there are no reactive phenomena such as inflammation or connective tissue proliferation, and only blood vessels’ dilatation can be noticed, along with numerous shapes (karyorrhexis, karyopycnosis, apoptotic bodies) of apoptotic cells. Notably, sham-operated and laser-irradiated mice reveal no noticeable brain damage ([23]; data not shown).

Our findings are consistent with prior studies demonstrating the high efficiency of PDT for C6 glioma in vivo, with glioma damage observed in deep brain regions [16]. A synthetic porphyrin compound, 2,4-(a43-dihydroxyethyl)deuteroporphyrin IX tetrakiscarborane carboxylate ester (BOPP), was used in this study [16,20]. Other porphyrin-based photosensitizers (e.g., Photofrin) have been applied for PDT of brain tumors in rats [24] and in clinical studies [25]. Similarly, chlorin-based photosensitizers (Photolon, Talaporfin) have also been tested [26,27]. However, none has yet reached clinical approval for brain malignancies [2,3,28]. 

A single dose (40 mg ETPA/kg mouse body weight) was used in our study. This drug dose regime in our mouse model was chosen following prior studies on PDT in rat models of brain tumors (keeping in mind the drug dose interspecies conversion rate [29]). The dose consisted of 2.5 mg/kg for Photolon [26], 25 mg/kg for BOPP [16], 40 mg/kg for hematoporphyrin derivative [30], or 100 mg/kg for 5-aminolevulinic acid [31]. The purpose of our work was not to find optimal concentrations/conditions for PDT but rather to provide a proof-of-concept demonstration of the potential of a natural chlorin—ETPA from *O. sarsii*—to provide photodynamic damage to the brain tumor. It is worth noting that we chose a low illumination regime (10 times less, as compared with [16]) to avoid thermal damage to the brain. Despite this sparing regime, we managed to achieve necrotic death within the irradiated area, indicating the high potential of ETPA for PDT.

## 4. Discussion

Porphyrin derivatives as unique molecules showing powerful phototherapeutic effects have found applications in anticancer PDT [4]. In our previous work, a new natural chlorin from the Pacific brittle star *Ophiura sarsii* was discovered [10]. In this current work, we uncovered multiple novel elements to the biology of this compound, (3*S*,4*S*)-14-Ethyl-9-(hydroxymethyl)-4,8,13,18-tetramethyl-20-oxo-3-phorbinepropanoic acid (ETPA) and its development toward PDT applications. 

Although known in some marine invertebrates including some Echinodermata such as the sea urchin *Strongylocentrotus purpuratus* [8], porphyrins were considered absent in the class of Ophiuroidea [11]. Thus, following our discovery of ETPA in *Ophiura sarsii*, we hypothesized that this chlorin compound could be derived from a food source—perhaps a seasonal one—of these brittle stars [10]. However, our current data unequivocally demonstrate that ETPA is endogenous to this marine invertebrate. Subsequent research might be directed to the delineation of the biosynthetic routes of this first-ever Ophiuroidea porphyrin. Porphyrin biosynthesis has been well studied, with one of the conserved enzymes, aminolevulinic acid (ALA) synthase, producing the key intermediate 5-aminolevulinic acid from glycine and succinyl–CoA [8]. The *Strongylocentrotus purpuratus* genome encodes an ALA synthase 62% identical to the mouse ortholog [8,32]. ALA synthase has been cloned from other marine invertebrates such as the bivalve *Patinopecten yessoensis*, and conserved gene sequences have been identified across taxonomic groups [33]. It is thus conceivable that despite the lack of the brittle star *O. sarsii* genomic data, its ALA synthase could be cloned in the future, along with the genes encoding other components of the porphyrin biosynthetic route. Further investigations could also be directed to the question of the role ETPA plays in the brittle star. As a major metabolite that can be isolated in large quantities, this chlorin compound is likely to play an important biological role in *O. sarsii*, such as participation in the electron transfer processes or protection from predators.

To investigate the medical utility of ETPA, we here performed proof-of-concept PDT studies in vitro and in vivo. The former assessed the phototoxic properties of ETPA against a panel of breast cancer and glioma cell lines, revealing sub-micromolar efficiency upon red-light irradiation. The latter relied on a popular animal model of glioblastoma demonstrating a remarkable PDT effect. Six hours post IV injection of ETPA, targeted red laser irradiation produced a dramatic photoablation in the brain tumor, leading to glioma necrosis. Notably, in the course of our experimentation, we did not observe any acute toxicity in the mice due to IV injection of ETPA, agreeing with studies with other photosensitizers on the good tolerability (and brain barrier permeability) of this group of compounds (e.g., [16,20]). More detailed pharmacokinetics and pharmacodynamics studies will be performed in the future, along with the broader assessment of the laser irradiation protocols, to further optimize the applicability of ETPA for the treatment of brain tumors (and other tumors) in animal models. A modification of the method can also be conceivable using fiber optic implantation into deeper brain regions to achieve maximal and maximally focused irradiation in the desired area [34]. Such future developments could be promising given the fact that none of the currently available photosensitizers has yet been approved for brain tumor PDT [2,3] despite several reaching clinical studies [25,27,28].

The abundance of ETPA in *O. sarsii* and the ease of its purification, along with its promising applications in PDT, make it attractive to consider upscaling of production of this natural product. Its abundance in its host is multiplied by the abundance of this brittle star species, wide-spread from Northern Atlantic, over the Arctic, and all the way to Northern Pacific, inhabiting waters from shallow to deep [35,36]. These features prompt considering mariculture of *O. sarsii* as has proven successful for other North Pacific invertebrates [37]. These possibilities, along with the preclinical developments of ETPA for PDT applications, may lead to the emergence of natural product-based novel photodynamic therapeutics.

## Data Availability

The data presented in this study are fully available in the main text and supplementary materials of this article.

## Supplementary Materials

The following are available online at https://www.mdpi.com/article/10.3390/biomedicines10010134/s1, Supplementary Figure S1: Mechanism of photosensitized DPIBF oxygenation, Supplementary Figure S2: Mechanism of photosensitized IR phosphorescence of singlet oxygen, Supplementary Figure S3: Absorption spectrum of ETPA in acetone, Supplementary Figure S4: Dark cytotoxicity of ETPA against C6 and HEK-293 cells.
