# Supplementary material for: Chlorin Endogenous to the North Pacific Brittle Star Ophiura sarsii for Photodynamic Therapy Applications in Breast Cancer and Glioblastoma Models
Source: Biomedicines. 2022 Jan 8;10(1):134. doi: 10.3390/biomedicines10010134 (PMC8773836; doi:10.3390/biomedicines10010134)
Supplement: Supplementary file 1 [file biomedicines-10-00134-s001.zip › biomedicines-1515544-supplementary.pdf]

Chlorin endogenous to the North Pacific brittle star *Ophiura sarsii* for photodynamic therapy applications in breast cancer and glioblastoma models

Antonina Klimenko, Elvira E. Rodina, Denis Silachev, Maria Begun, Valentina A. Babenko, Anton S. Benditkis, Anton S. Kozlov, Alexander A. Krasnovsky, Yuri S. Khotimchenko, and Vladimir L. Katanaev

Supplementary Materials

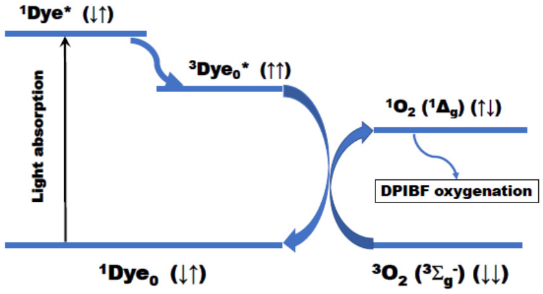

Supplementary Figure S1. Mechanism of photosensitized DPIBF oxygenation. ^{1}Dye$_0$, ^{1}Dye* and ^{3}Dye* are molecules of photosensitizers in the ground and excited singlet and triplet states; $^3O_2$ and $^1O_2$ are oxygen molecules in the ground and singlet states.

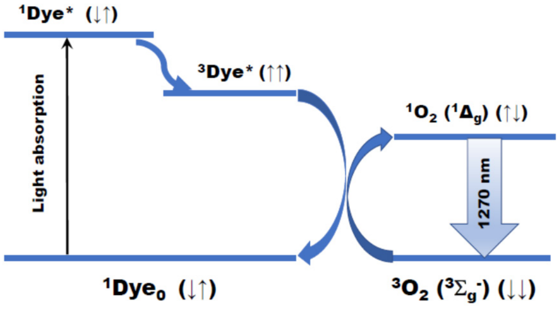

Supplementary Figure S2. Mechanism of photosensitized IR phosphorescence of singlet oxygen. ^{1}Dye$_0$, ^{1}Dye* and ^{3}Dye* are molecules of photosensitizer pigments in the ground and excited singlet and triplet states; $^3O_2$ and $^1O_2$ are oxygen molecules in the ground and singlet states.

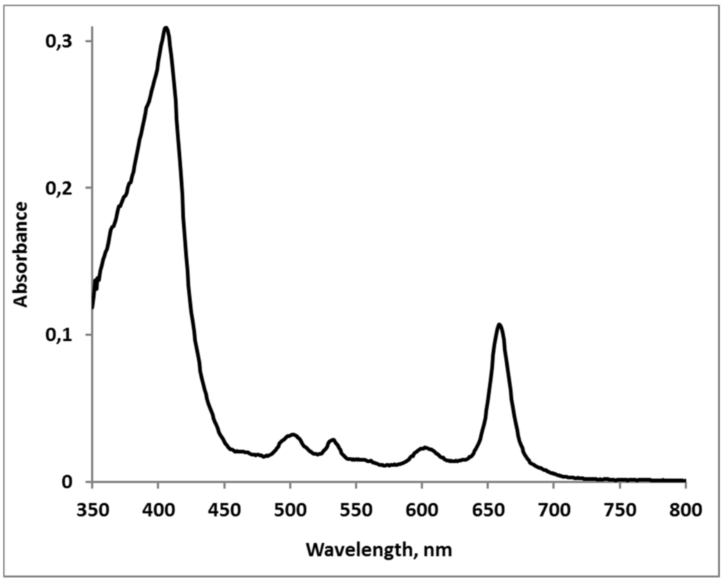

Supplementary Figure S3. Absorption spectrum of ETPA in acetone.

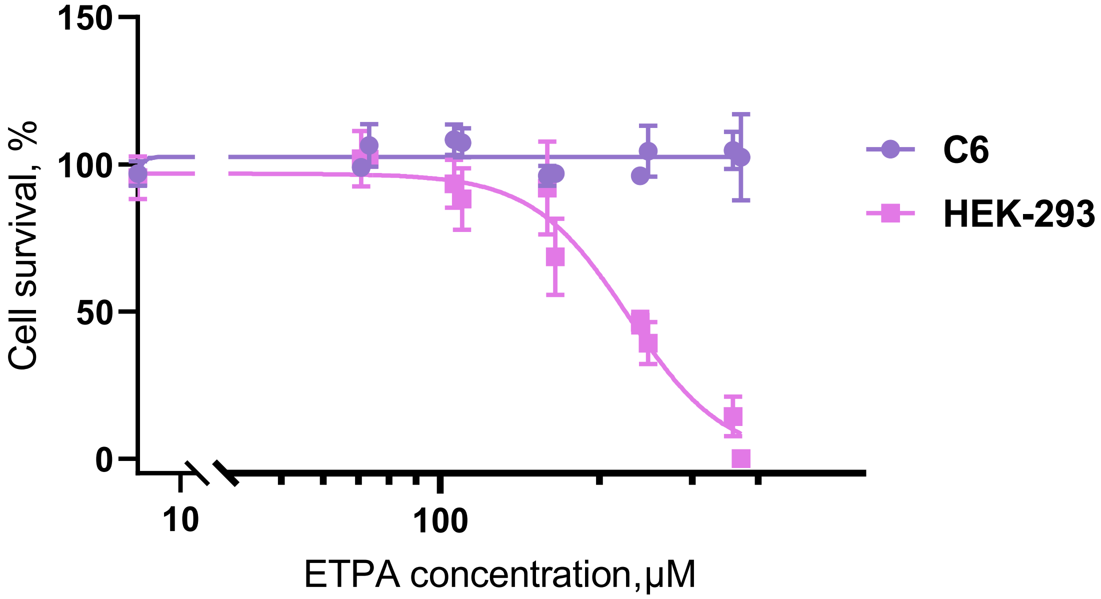

Supplementary Figure S4. Dark cytotoxicity of ETPA against C6 and HEK-293 cells. ETPA was cytotoxic in the dark against HEK-293 cells with the IC_{50} of 226 ± 17µM (mean ± sd, n=3). In contrast, no cytotoxicity in the dark could be reached against the rat glioma C6 cells at the highest tested concentrations of 400µM ETPA.
